# Effect of Clinical Rotations in Domiciliary Dental Care on Dental Students' Attitudes, Empathy, and Perceptions Towards Institutionalized Older Adults in Zurich in Switzerland

**DOI:** 10.1111/scd.70192

**Published:** 2026-05-21

**Authors:** Angela Stillhart, Mark Solomons, Claudio R. Leles, Hansmartin Spatzier, Murali Srinivasan

**Affiliations:** ^1^ Clinic For General‐, Special Care‐ and Geriatric Dentistry, Center For Dental Medicine University of Zurich Zurich Switzerland; ^2^ Faculty of Dentistry Federal University of Goias Goiania Brazil; ^3^ Center of Excellence in Precision Medicine and Digital Health, Geriatric Dentistry and Special Patients Care International Program, Department of Physiology, Faculty of Dentistry Chulalongkorn University Bangkok Thailand

**Keywords:** attitudes/opinion surveys, domiciliary dental care, gerodontology, geriatric dentistry, institutional dental care, older adults, oral health

## Abstract

**Objective:**

This study evaluated the impact of structured clinical rotations in domiciliary dental care (DDC) on undergraduate dental students’ attitudes, empathy, and perceptions toward institutionalized older adults in Zurich, Switzerland.

**Methods and results:**

Eighty‐one dental students (mean age: 28.1 ± 5.9) participated in one to four DDC rotations within institutional care settings. Pre‐ and post‐rotation assessments used four validated instruments: the UCLA Geriatric Attitude Scale (GAS‐14), Dementia Attitude Scale (DAS), Ageing Semantic Differential Scale (ASDS), and Modified Maxwell‐Sullivan Attitude Scale (M‐MSAS). Data were analyzed using non‐parametric tests and Bonferroni‐adjusted comparisons (*α* = 0.05). Students with one rotation had significantly higher GAS‐14 scores (3.49 ± 0.44) than those with two (3.19 ± 0.38, *p* = 0.001). Empathy (M‐MSAS) increased with more exposure, peaking at four visits (11.05 ± 1.18, *p* = 0.021). ASDS scores indicated more negative perceptions after two visits (132.20 ± 12.87) compared to one (122.59 ± 15.26, *p* = 0.009). No significant change in dementia‐related knowledge was found (*p* = 0.251).

**Conclusion:**

Structured clinical rotations significantly enhanced dental students' attitudes, empathy, and perceptions toward elderly care, highlighting variations based on the frequency of clinical exposure.

## 1 Introduction

The rapid increase in global life expectancy has led to a significant demographic shift characterized by a growing elderly population. According to recent projections, the population aged 65 years and older will nearly double within the next three decades, posing considerable public health implications [1, 2]. Among these concerns, oral health emerges as a critical component that profoundly affects the overall health, nutritional status, quality of life, and psychosocial well‐being of older adults [3, 4, 5]. Consequently, dentists' preparedness to effectively address the oral health care needs of this demographic group becomes paramount. Providing optimal dental care for elderly patients requires specialized clinical knowledge and the development of favorable attitudes, empathic communication, and heightened sensitivity towards the challenges older adults face [6]. Despite the growing relevance and necessity for geriatric dentistry, evidence suggests that dental professionals often demonstrate inadequate training and insufficient readiness in managing older patients [7]. The limited exposure of dental students to elderly patients during their undergraduate training may negatively influence their confidence, empathy, and willingness to care for the elderly population [8]. This phenomenon presents a significant gap in dental education that must be urgently addressed.

Attitudes and empathy among healthcare providers play critical roles in clinical outcomes and patient satisfaction [9, 10]. Specifically, dentists with higher empathy and positive attitudes towards elderly patients are associated with better clinical interactions, improved patient compliance, and higher patient satisfaction and trust [11]. Yet, studies consistently highlight deficiencies in dental education regarding geriatric dentistry [8]. Often, curriculum emphasis on this field remains limited, contributing to inadequate exposure and diminished interest among future dental professionals in elderly patient care [12]. Thus, exploring and bridging this educational gap becomes crucial, particularly considering the expanding geriatric patient population and the complexities of treating older adults.

Hands‐on clinical exposure in geriatric care settings has been proposed as a key factor capable of significantly shaping dental students' professional attitudes, perceptions, and empathy towards older patients [13, 14, 15, 16]. It is hypothesized that direct clinical interactions with elderly patients, especially within community or nursing home settings, positively impact students' attitudes, empathy, and perceptions about older patient management [17]. However, empirical evidence to robustly substantiate these assumptions is limited and fragmented, and warrants further systematic investigation.

Evaluating dental students' perceptions and attitudes through validated psychometric tools offers scientific rigor, objectivity, and comparability of outcomes. Instruments such as the 14‐item UCLA Geriatric Attitudes Survey (GAS‐14) [18], Aging Semantic Differential Scale (ASDS‐32) [19, 20], Modified Maxwell Sullivan Attitudes Scale [21], Dementia Attitude Scale [22], have demonstrated reliability and validity in various health professional populations. Such instruments facilitate objective quantification of students' empathy levels, attitudes, and perceptions about elderly patients, providing valuable baseline and outcome measures for educational interventions [23, 24, 25]. Nevertheless, studies have systematically assessed changes in dental students' empathy, opinions, and attitudes specifically following structured clinical rotations focused exclusively on geriatric dentistry [26].

Limited information exists regarding how structured geriatric clinical exposure during dental training affects students' empathy, opinions, and overall attitudes towards older adult patients. Furthermore, few studies have critically assessed the educational impact of hands‐on geriatric rotations within residential or nursing home settings, where elderly individuals often present with complex medical, cognitive, psychological, and functional challenges. Clarifying these gaps will yield insights into the efficacy of targeted educational strategies and inform curricular improvements tailored to geriatric dentistry.

This study aimed to evaluate the influence of structured hands‐on clinical training in a geriatric home setting on dental students' attitudes, empathy, and perceptions toward elderly patient care. By employing validated questionnaires administered before and after clinical rotation, this research sought to provide evidence on how direct exposure to elderly patient care shaped dental students' professional development. Identifying changes in students' empathy and attitudes holds significance beyond educational contexts, potentially influencing long‐term clinical behaviors, quality of patient‐dentist relationships, and ultimately, patient outcomes among the elderly. Findings from this study may guide evidence‐based curricular reforms that enhance dental students' preparedness, sensitivity, and capability to meet the complex oral health needs of an aging global population.

## 2 Materials and Methods

### 2.1 Ethics Approval

The study protocol was sent for ethics clarification to the relevant ethics board in the canton of Zurich, and the board decided that it did not require a formal ethics approval (KEK‐Zurich: BASEC‐ID Req‐2024‐01558). Each student received detailed information regarding the study objectives, procedures, confidentiality, and voluntary participation. Informed consent was obtained from all students prior to their participation. The confidentiality of participants' data was rigorously maintained, and no identifiable personal information was disclosed in the analysis or publication of results.

### 2.2 Participants

The study recruited final year dental students in the Center for Dental Medicine at the University of Zurich. All students had completed standardized theoretical coursework in geriatric dentistry before commencing clinical rotations in the domiciliary dental care service of the Clinic of General, Special care and Geriatric Dentistry in the Center for Dental Medicine, at the University of Zurich, in Zurich, Switzerland.

### 2.3 Sample Size

A post‐hoc sample size calculation was performed based on the difference between two dependent means (matched pairs) for a sample size of 60 subjects and previous literature indicating moderate effect sizes for educational interventions in attitudes and empathy toward elderly patient care. Therefore, using an alpha level of 0.05 and assuming a moderate effect size (Cohen's *d* = 0.5), a high power <0.95 was achieved. To account for potential dropout or students’ reluctance to participate (approximately 10%), the final sample size was calculated as 66. The final sample size was rounded to 80 students, corresponding to the number of final year students of two successive academic years. The sample size calculation was done using free software (G*Power, version 3.1.9.6, Düsseldorf, Germany) [27, 28].

### 2.4 Survey Questionnaires

Four validated psychometric instruments and a validated general questionnaire were employed to measure participants' attitudes, empathy, and perceptions toward elderly patients. These instruments were carefully selected due to their established reliability, validity, and applicability in geriatric education research:

1. **Geriatric Attitude Scale (GAS‐14)** [18]: The GAS is a unidimensional, standardized, validated 14‐item questionnaire assessing healthcare professionals' attitudes and willingness toward geriatric patient management. Responses are rated on a five‐point Likert scale, with higher scores indicating more positive attitudes toward elderly patient care and management.
2. **Dementia Attitude Scale (DAS)** [22]: DAS is a validated 20‐item scale specifically designed to measure healthcare professionals’ attitudes toward dementia patients. It evaluates comfort, compassion, and cognitive biases toward dementia care, scored using a Likert‐type scale. The scale has a two‐factor structure, labeled “dementia knowledge” (10 items) and “social comfort” (10 items). Higher scores represent more positive and empathetic attitudes toward dementia patients.
3. **Modified Maxwell‐Sullivan Attitudes Survey (M‐MSAS)** [21]: The M‐MSAS is a validated questionnaire used to assess empathy, personal comfort, and willingness to interact with elderly patients. It consists of 28 items scored on a Likert‐type scale, with higher total scores indicating greater empathy and comfort with elderly patient interactions.
4. **Ageing Semantic Differential Survey (ASDS) [** 19, 20]: The ASDS is a validated survey tool that measures perceptions and stereotypical attitudes toward aging. It comprises of 32 bipolar adjective pairs (e.g., active‐passive, independent‐dependent), rated on a semantic differential scale. The scale comprises four factors: Factor 1 (F1): Instrumentality, Factor 2 (F2): Autonomy, Factor 3 (F3): Acceptability, and Factor 4 (F4): Integrity. Lower scores represent more positive perceptions about older adults and aging.
5. **Specialized Purpose‐Built Questionnaire (SQ) [** 13, 14, 15]: This questionnaire comprised a set of 9 questions that specifically evaluate the students' opinions on the importance and adequacy of geriatric dentistry in their present curriculum. It has been validated in multiple previous studies.

All questionnaires were translated from their original English versions into German while adhering to the good practice guidelines for translation of patient reported outcomes [29]. Translation and linguistic validation were rigorously conducted by bilingual translators using forward and backward translation methods.

### 2.5 Study Protocol

This study utilized repeated measures. Students completed the questionnaires at two distinct time points: immediately prior to initiating each clinical rotation (baseline assessment) and immediately after completing each rotation (post‐rotation assessment). Assessments occurred once, before and after their single clinical rotation or each time preceding and following every clinical rotation for those students who participated in multiple rotations, enabling analysis of cumulative changes in attitudes and empathy.

Clinical rotations included structured hands‐on clinical experiences within geriatric residential care facilities. Under close supervision from trained dental faculty specializing in geriatric dentistry, students performed clinical examinations, preventive and therapeutic procedures, oral health education, and management of elderly patients, including those with cognitive impairments, functional disabilities, and complex medical histories. Each rotation provided the students with exposure to geriatric dental care in real clinical settings.

### 2.6 Statistical Analysis

Descriptive statistics, including means, standard deviations, medians, frequencies, and percentages, were calculated to summarize baseline characteristics and questionnaire responses. Normality of the data was not confirmed by the Kolmogorov–Smirnov test (*p* < 0.05) prior to conducting parametric tests. Non‐parametric tests, Mann–Whitney U and Kruskal–Wallis tests were performed accordingly, and post hoc Bonferroni tests were applied with the alpha level set to 0.05. All statistical analyses were performed using a statistical software (IBM SPSS Statistics for Mac, version 30.0, IBM Corp., Armonk, New York, USA).

## 3 Results

### 3.1 Attitudes Toward Older Adults

Table 1 summarizes students' attitudes toward treating older adults measured by the UCLA Geriatric Attitude Scale (GAS‐14) following clinical rotations in domiciliary dental care (DDC). A total of 81 students with a mean age of 28.1 years (± 5.9) participated with varying rotation exposure: 39 students (1 visit), 14 students (2 visits), 21 students (3 visits), and 7 students (4 visits). Majority of the participants were women (60.5%). The overall mean GAS‐14 score was 3.35 (± 0.44). Statistical analysis indicated a significant difference among groups (*p* = 0.002). Pairwise comparisons revealed significantly higher GAS‐14 scores after 1 visit compared to 2 visits (*p* < 0.001). However, comparisons between other groups (e.g., 3 visits vs. 4 visits, 4 visits vs. 1 visit) did not show statistically significant differences after Bonferroni adjustment.

**TABLE 1:** Students' attitudes towards treating older adults assessed with the 14‐item UCLA Geriatric Attitude Scale (GAS‐14) after clinical rotations in institutions while delivering care with a domiciliary dental service unit.

DDC visits (*n*)	Students (*n*)	Mean	SD	SE	95%CI		*p*‐value^a^
					Lower	Upper	
1	39	3.49	0.44	0.05	3.39	3.59	
2	14	3.19	0.38	0.05	3.09	3.30	
3	21	3.33	0.42	0.04	3.25	3.40	
4	7	3.35	0.49	0.07	3.22	3.49	
Total	81	3.35	0.44	0.02	3.30	3.40	0.002
							
**Pairwise comparison**			Test statistic	SE	Std. Test statistic	Sig.	Adj. Sig.^b^
2 visits versus 4 visits			−27.982	17.24	−1.623	0.105	0.628
2 visits versus 3 visits			−29.671	14.66	−2.025	0.043	0.257
2 visits versus 1 visit			61.622	15.98	3.856	<.001	0.001
4 visits versus 3 visits			1.688	14.66	0.115	0.908	1.000
4 visits versus 1 visit			33.640	15.98	2.105	0.04	0.212
3 visits versus 1 visit			31.951	13.15	2.430	0.02	0.090

Abbreviation: DDC, Domiciliary Dental Care visits; n, number; SD, standard deviation; SE, standard error; 95% CI, 95 % confidence interval.

^a^Mann–Whitney U test.

^b^Bonferroni adjusted *p*‐value for Kruskal–Wallis multiple comparison test; Significance: *p* < 0.05

### 3.2 Knowledge and Comfort about Dementia

Table 2 illustrates students' comfort and knowledge regarding dementia, assessed using the Dementia Attitude Scale (DAS). The overall mean score for Social Comfort was 43.73 (± 5.31), Dementia Knowledge was 82.65 (± 10.19), and the Overall DAS was 95.92 (± 9.85). No statistically significant differences were observed between groups for Social Comfort (*p* = 0.324), Dementia Knowledge (*p* = 0.386), or Overall DAS scores (*p* = 0.251), indicating consistent comfort levels and dementia knowledge among students regardless of the number of visits.

**TABLE 2:** Assessment of the students' knowledge and comfort about adults with dementia assessed with the Dementia Attitude Scale (DAS) after clinical rotations in institutions while delivering care with a domiciliary dental service unit.

Domains	DDC visits (*n*)	Students (*n*)	Mean	SD	SE	95%CI		*p*‐value^a^
						Lower	Upper	
Social comfort	1	39.00	44.62	5.82	0.66	43.30	45.93	
	2	14.00	43.64	5.63	0.75	42.14	45.15	
	3	21.00	43.25	5.37	0.48	42.30	44.20	
	4	7.00	43.64	3.90	0.52	42.60	44.69	
	Total	81.00	43.73	5.31	0.30	43.14	44.32	0.324
Dementia knowledge	1	39.00	84.27	11.23	1.27	81.74	86.80	
	2	14.00	82.50	10.82	1.45	79.60	85.40	
	3	21.00	81.78	10.37	0.93	79.94	83.61	
	4	7.00	82.50	7.20	0.96	80.57	84.43	
	Total	81.00	82.65	10.19	0.57	81.52	83.78	0.386
Overall DAS	1	39.00	97.68	10.40	1.18	95.33	100.02	
	2	14.00	94.80	8.66	1.16	92.48	97.12	
	3	21.00	95.40	9.97	0.89	93.64	97.17	
	4	7.00	95.77	9.82	1.31	93.14	98.40	
	Total	81.00	95.92	9.85	0.55	94.83	97.02	0.251

DDC: Domiciliary Dental Care visits; n: number; SD: standard deviation; SE: standard error; 95% CI: 95 % confidence interval.

^a^Mann–Whitney U test; Significance: *p* < 0.05

### 3.3 Perceptions and Stereotypes About Aging

Students' perceptions and stereotypical attitudes toward aging, assessed with the Aging Semantic Differential Scale (ASDS), are presented in Table 3. The overall mean ASDS score was 127.27 (± 14.93). Statistical analysis indicated significant differences among the groups (*p* = 0.014). Specifically, pairwise comparisons demonstrated that students with 2 visits had significantly higher (less positive) overall ASDS scores compared to those with only 1 visit (*p* = 0.002). No other significant pairwise differences were noted after Bonferroni correction. Notably, significant differences emerged in the Acceptability (*p* < 0.001) and Integrity (*p* = 0.013) domains, suggesting varied perceptions based on the extent of clinical rotation experience.

**TABLE 3:** Evaluation of the students' opinions about older adults assessed with the the Ageing Semantic Differental Scale (ASDS) after clinical rotations in institutions while delivering care with a domiciliary dental service unit.

Domains	DDC visits (*n*)	Students (*n*)	Mean	SD	SE	95%CI		*p*‐value^a^	
						Lower	Upper	
Factor 1: Instrumentality	1	39.00	24.05	3.70	0.42	23.22	24.89	
	2	14.00	25.61	3.43	0.46	24.69	26.52	
	3	21.00	25.01	3.36	0.30	24.42	25.60	
	4	7.00	24.88	3.23	0.43	24.01	25.74	
	Total	81.00	24.85	3.46	0.19	24.47	25.24	0.298
Factor 2: Autonomy	1	39.00	31.97	4.21	0.48	31.02	32.92	
	2	14.00	33.57	4.20	0.56	32.45	34.70	
	3	21.00	33.47	4.66	0.42	32.65	34.29	
	4	7.00	33.20	4.12	0.55	32.09	34.30	
	Total	81.00	33.07	4.41	0.25	32.58	33.56	0.108
Factor 3: Acceptability	1	39.00	23.82	5.16	0.58	22.66	24.98	
	2	14.00	27.48	3.36	0.45	26.58	28.38	
	3	21.00	25.65	3.91	0.35	24.96	26.34	
	4	7.00	24.59	5.44	0.73	23.13	26.05	
	Total	81.00	25.34	4.61	0.26	24.83	25.85	**<0.001**
Factor 4: Integrity	1	39.00	18.44	3.50	0.40	17.65	19.22	
	2	14.00	20.57	2.76	0.37	19.83	21.31	
	3	21.00	19.60	3.52	0.31	18.98	20.22	
	4	7.00	19.39	2.96	0.40	18.60	20.19	
	Total	81.00	19.45	3.35	0.19	19.08	19.82	**0.013**
Overall ASDS	1	39.00	122.59	15.26	1.73	119.15	126.03	
	2	14.00	132.20	12.87	1.72	128.75	135.64	
	3	21.00	128.02	14.70	1.31	125.43	130.61	
	4	7.00	127.18	15.40	2.06	123.06	131.30	
	Total	81.00	127.27	14.93	0.84	125.62	128.92	**0.014**
**Pairwise comparison**									
Overall ASDS				Test statistic	SE	Std. Test statistic	Sig.	Adj. Sig.^b^
1 visit versus 4 visits				−19.680	15.993	−1.231	0.218	1.000
1 visit versus 3 visits				−29.096	13.155	−2.212	0.027	0.162
1 visit versus 2 visits				−50.644	15.993	−3.167	0.002	**0.009**
4 visits versus 3 visits				9.416	14.665	0.642	0.521	1.000
4 visits versus 2 visits				30.964	17.256	1.794	0.073	0.436
3 visit versus 2 visits				21.549	14.665	1.469	0.142	0.850

Abbreviation: DDC, Domiciliary Dental Care visits; *n*, number; SD, standard deviation; SE, standard error; 95% CI, 95 % confidence interval.

^a^Mann–Whitney U test.

^b^Bonferroni adjusted *p*‐value for Kruskal–Wallis multiple comparison test; Significance: *p* < 0.05

### 3.4 Empathy Toward Older Adults

Empathy levels were assessed using the Modified Maxwell‐Sullivan Attitude Scale (M‐MSAS), with results summarized in Table 4. The mean Overall M‐MSAS score across all students was 34.43 (± 2.93), with statistically significant differences across groups (*p* = 0.045). The Empathy domain demonstrated significant variations (*p* = 0.014), notably showing that students with 4 visits exhibited significantly higher empathy scores compared to students with only 2 visits (*p* = 0.004), although this was the only significant pairwise difference after Bonferroni adjustment. The Attitude domain did not significantly differ among groups (*p* = 0.378).

**TABLE 4:** Assessment of the students' empathy towards older adults assessed with the modified Maxwell‐Sullivan Attitude Scale (M‐MSAS) after clinical rotations in institutions while delivering care with a domiciliary dental service unit.

Domains	DDC visits (*n*)	Students (*n*)	Mean	SD	SE	95%CI		*p*‐value^a^
						Lower	Upper	
Attitude	1	39.00	23.81	1.92	0.22	23.37	24.24	
	2	14.00	23.89	1.91	0.26	23.38	24.41	
	3	21.00	23.62	2.00	0.18	23.27	23.97	
	4	7.00	24.14	2.04	0.27	23.60	24.69	
	Total	81.00	23.81	1.97	0.11	23.59	24.03	0.378
Empathy	1	39.00	10.91	1.25	0.14	10.63	11.19	
	2	14.00	10.20	1.77	0.24	9.72	10.67	
	3	21.00	10.44	1.90	0.17	10.10	10.77	
	4	7.00	11.05	1.18	0.16	10.74	11.37	
	Total	81.00	10.62	1.65	0.09	10.44	10.80	**0.014**
Overall M‐MSAS	1	39.00	34.72	2.62	0.30	34.13	35.31	
	2	14.00	34.09	2.94	0.39	33.30	34.88	
	3	21.00	34.06	3.11	0.28	33.51	34.60	
	4	7.00	35.20	2.75	0.37	34.46	35.93	
	Total	81.00	34.43	2.93	0.16	34.10	34.75	0.045
**Pairwise comparison**								
Empathy				Test Statistic	Std. Error	Std. Test Statistic	Sig.	Adj. Sig [b].
2 visits versus 3 visits				−17.068	14.342	−1.190	0.234	1.000
2 visits versus 1 visit				35.828	15.641	2.291	0.022	0.132
2 visits versus 4 visits				−49.188	16.876	−2.915	0.004	**0.021**
3 visits versus 1 visit				18.759	12.866	1.458	0.145	0.869
3 visits versus 4 visits				−32.119	14.342	−2.239	0.025	0.151
1 visit versus 4 visits				−13.360	15.641	−0.854	0.393	1.000

Abbreviation: DDC, Domiciliary Dental Care visits; n, number; SD, standard deviation; SE, standard error; 95% CI, 95 % confidence interval.

^a^Mann–Whitney U test.

^b^Bonferroni adjusted *p*‐value for Kruskal–Wallis multiple comparison test; Significance: *p* < 0.05

### 3.5 Opinions on Geriatric Dentistry Curriculum

Table 5 provides students' evaluations of the geriatric dentistry curriculum. The majority strongly agreed that geriatric dentistry is crucial (56.8%) and that mobile dental clinics effectively addressed elderly care (84.0%). Approximately 69.2% agreed (34.6% somewhat and 34.6% strongly) that the current geriatric curriculum was adequate. 49.3% expressed confidence in independently managing elderly patients. Preferences indicated a stronger inclination toward clinic‐based settings (54.3% agreement) over hospital or nursing home settings, highlighting dental students' specific comfort and learning preferences.

**TABLE 5:** Students' opinions of the current geriatric curriculum, confidence and preferences in providing care to elderly patients.

Supplementary questions (SQ)	Strongly disagree [*n* (%)]	Somewhat disagree [*n* (%)]	Neutral [*n* (%)]	Somewhat agree [*n* (%)]	Strongly agree [*n* (%)]
SQ1: Geriatric dentistry is an important aspect of the dental curriculum	0 (0)	4 (4.9)	5 (6.2)	26 (32.1)	46 (56.8)
SQ2: The geriatric program in the teaching curriculum is adequate	1 (1.2)	8 (9.9)	16 (19.8)	28 (34.6)	28 (34.6)
SQ3: I am confident in treating the elderly patient on my own independently	5 (6.2)	22 27.2)	14 (17.3)	36 (44.4)	4 (4.9)
SQ4: I am not confident in treating the elderly patient unsupervised	13 (16.0)	23 (28.4)	22 (27.2)	21 (25.9)	2 (2.5)
SQ5: I would like to have hands‐on experience in treating elderly patients during my undergraduate curriculum	0 (0)	5 (6.2)	15 (18.5)	31 (38.3)	30 (37.0)
SQ6: I would prefer to treat the elderly patients in a clinic setting	0 (0)	7 (8.6)	30 (37.0)	35 (43.2)	9 (11.1)
SQ7: I would prefer to treat the elderly patients in a hospital setting	1 (1.2)	18 (22.2)	39 (48.1)	16 (19.8)	7 (8.6)
SQ8: I would prefer to treat the elderly patients in nursing home setting	2 (2.5)	14 (17.3)	42 (51.9)	16 (19.8)	7 (8.6)
SQ9: Mobile dental clinics are a good solution for elders without access to care	0	0	2 (2.5)	11 (13.6)	68 (84.0)

Abbreviation: *n*, number; %, percentage

## 4 Discussion

This study evaluated the impact of structured clinical rotations in domiciliary dental care (DDC) on dental students' attitudes, empathy, perceptions, and comfort regarding the management of institutionalized older adults. Findings from this study contribute valuable insights into the educational value of targeted geriatric clinical experiences, underscoring important implications for dental curricula and professional development.

An essential finding was the significant improvement in the students' attitudes towards older adults, measured by the UCLA Geriatric Attitude Scale (GAS‐14), particularly after their initial DDC experience. Interestingly, students who participated in only one clinical rotation exhibited significantly more positive attitudes compared to those with two rotations. This outcome suggested that the initial clinical exposure profoundly impacted students’ attitudes, possibly reflecting a novelty effect or heightened enthusiasm during the first encounter. Conversely, repeated rotations did not incrementally enhance attitudes, potentially indicating a plateau effect. This observation aligns with previous studies showing diminishing attitudinal changes after initial positive educational experiences [13, 14, 15]. The implication is that while initial exposure is critical in fostering positive attitudes, maintaining these attitudes might require varied or more intense educational interventions, rather than merely repeated similar experiences.

Furthermore, the Aging Semantic Differential Scale (ASDS) revealed significant variations, notably in domains of Acceptability and Integrity. Students who completed two visits exhibited less favorable perceptions of older adults than those who had just one visit. This paradoxical finding might reflect increased awareness of the complexities and the challenges associated with elderly care observed during subsequent visits. Initially, idealized views might be tempered by realistic exposure to the difficulties older patients face, including cognitive impairments and functional disabilities, resulting in more nuanced, albeit less idealized, perceptions. Previous studies have suggested that realism in clinical exposure can alter initially idealistic perceptions among health professionals, highlighting the complexity of sustained positive attitudes [19, 20].

Empathy scores significantly differed based on rotation frequency. Students with more extensive experience (four rotations) showed significantly higher empathy than students with moderate exposure (two rotations). This significant finding underscored the cumulative benefit of repeated clinical experiences on empathy development. Empathy, crucial in patient‐centered care, enhances clinical interactions and patient satisfaction [10, 11]. Hence, repeated exposure in authentic clinical environments, despite potentially diminishing returns in attitudinal gains, appears necessary for empathy maturation. This finding corroborated prior research indicating empathy's responsiveness to repeated, meaningful clinical encounters [16, 23, 24]. Thus, dental curricula should consider structured repeated clinical interactions to bolster empathy, a critical competency in geriatric dentistry.

However, students' dementia‐related comfort and knowledge did not significantly differ across rotation frequencies. The uniformly high DAS scores indicated that baseline education in dementia care was effective, and short‐term clinical rotations may offer limited incremental value in this domain. This highlighted that foundational theoretical education effectively prepared students for dementia care, suggesting a need to differentiate objectives between foundational knowledge versus clinical attitudinal and empathy‐related goals within geriatric training programs.

A notable strength of this study was its methodological rigor. Employing validated psychometric instruments such as GAS‐14, DAS, ASDS, and M‐MSAS, this study provided robust quantitative measures of students' attitudes, empathy, and perceptions [13, 14, 15, 18, 19, 20, 21, 22]. Moreover, utilizing repeated measures allowed a precise evaluation of temporal changes following structured clinical exposure. Translation and linguistic validation of instruments into German also ensured reliability and cultural appropriateness for this Swiss student population.

Another salient feature was the inclusion of students with varying levels of clinical exposure (1–4 rotations). This provided unique insights into the incremental educational value of repeated exposure, revealing complex dynamics such as diminishing returns in attitude improvements but significant cumulative empathy development. Such nuanced insights are critical for designing effective geriatric curricula, highlighting that a tailored approach to geriatric clinical rotations may optimize educational outcomes in different domains.

Furthermore, assessing students' perceptions of the current curriculum and preferred clinical settings via the specialized questionnaire (SQ) offered valuable curricular feedback [4, 13, 14]. Most students strongly affirmed geriatric dentistry's importance and positively evaluated mobile dental units, indicating students’ appreciation for direct, hands‐on clinical engagement. However, confidence in independently managing older patients remained modest, signaling a gap between theoretical preparedness and perceived clinical competence. This discrepancy emphasized the need for enhanced hands‐on clinical experiences integrated throughout the dental training to bridge theory‐practice gaps effectively. Additionally, students preferred treating older patients in a clinic‐based setting rather than in nursing homes or in hospitals, indicating preferences for structured, controlled clinical environments. Understanding student preferences is essential to optimize learning environments, enhancing engagement and educational outcomes. Finally, the observational, prospective design and rigorous statistical analysis further strengthened the reliability and validity of findings. The study's power calculation ensured sufficient sample size, minimizing type II errors and enhancing the generalizability of results within similar educational contexts.

The significant and nuanced findings of this study have several practical implications for dental education. Firstly, dental curricula should incorporate early clinical rotations focused explicitly on geriatric populations, capitalizing on initial positive attitudinal gains. Simultaneously, curricula must integrate sustained and varied clinical exposure to facilitate continued empathy development without triggering potential attitudinal fatigue or negativity stemming from repeated exposure to clinical complexities. Future research should investigate longitudinal outcomes beyond the immediate post‐rotation assessments. Extended follow‐up studies could evaluate whether attitudinal and empathy gains translate into long‐term behavioral changes and improved patient care outcomes. Additionally, qualitative studies exploring students' subjective experiences and perceptions could complement quantitative findings, providing deeper insights into educational dynamics affecting attitudes and empathy development. Moreover, comparing different educational strategies (e.g., rotation duration, interdisciplinary teams, reflective learning) would elucidate optimal methods for enhancing student preparedness in geriatric dentistry. Understanding how curriculum adjustments directly impact professional competencies and patient care quality remains a critical area for future research.

To summarize, the findings of this study partially reject the null hypothesis, which posited that structured hands‐on clinical rotations in a geriatric home setting would have no effect on dental students’ attitudes, empathy, or perceptions toward elderly patients. Statistically significant improvements were observed in attitudes (GAS‐14), perceptions (ASDS), and empathy (M‐MSAS) following clinical rotations, particularly with repeated exposure. However, the hypothesis holds true for certain domains, such as dementia‐related knowledge and comfort (DAS), where no significant differences were found between rotation groups. These mixed results highlight the targeted and domain‐specific influence of clinical experiences on student development in geriatric dentistry.

## 5 Conclusions

Structured clinical rotations significantly enhanced dental students' attitudes, empathy, and perceptions toward elderly care, highlighting variations based on the frequency of clinical exposure. Overall, students positively valued geriatric dentistry within their curriculum, favoring hands‐on clinical experiences, particularly in mobile or clinical settings. The findings advocate for carefully balanced clinical exposure within dental curricula, strategically designed to maximize positive attitudinal shifts, empathy development, and clinical competencies essential for addressing the complex oral healthcare needs of aging populations.

## Funding

No funding was received for this research.

## Conflicts of Interest

The authors declare no conflicts of interest.

## Clinical Registration number

Not applicable
